# Psychometric evaluation of the Positive Mental Health (PMH) scale using item response theory

**DOI:** 10.1186/s12888-022-04162-0

**Published:** 2022-07-28

**Authors:** Lusine Vaganian, Maren Boecker, Sonja Bussmann, Michael Kusch, Hildegard Labouvie, Jürgen Margraf, Alexander L. Gerlach, Jan C. Cwik

**Affiliations:** 1 Department of Clinical Psychology and Psychotherapy, Faculty of Human Sciences, University of Cologne, Pohligstr. 1, 50969 Cologne, Germany; 2 Institute of Medical Psychology and Medical Sociology, University Hospital of RWTH Aachen, Aachen, Germany; 3 Child Neuropsychology Section, Department of Child and Adolescent Psychiatry, University Hospital of RWTH Aachen, Aachen, Germany; 4 Department I of Internal Medicine, Section: Clinical Psycho-Oncology, Working Group Psycho-Oncological Health Services Research, University Hospital of Cologne, Cologne, Germany; 5 Mental Health Research and Treatment Center, Faculty of Psychology, Ruhr-Universität Bochum, Bochum, Germany

**Keywords:** Mental health, Cancer, Rasch analysis, Psychometric properties, Well-being

## Abstract

**Background:**

The investigation of patient-reported outcomes and psycho-oncological interventions mainly focuses on psychological distress or psychopathology. However, the recognition of the equal importance of positive mental health (PMH) has increased lately. The PMH-scale is a brief questionnaire allowing to assess well-being in individuals in the general population and in patients. Previous studies evaluated the psychometric properties of the PMH-scale using classical test theory (CTT). This study is the first to investigate the PMH-scale in patients with cancer using item analysis according to the Rasch model.

**Methods:**

In total, *N* = 357 cancer patients participated in the study. A Rasch analysis of the PMH-scale was conducted including testing of unidimensionality, local independence, homogeneity and differential item functioning (DIF) with regard to age, gender, type of cancer, the presence of metastases, psycho-oncological support, and duration of disease. Additionally, the ordering of the item thresholds as well as the targeting of the scale were investigated.

**Results:**

After excluding one misfitting item and accounting for local dependence by forming superitems, a satisfactory overall fit to the Rasch model was established (χ^2^ = 30.34, *p* = 0.21). The new PMH-8 scale proved to be unidimensional, and homogeneity of the scale could be inferred. All items showed ordered thresholds, there was no further item misfit. DIF was found for age, but as the impact of DIF was not substantial, no adjustment related to the age-DIF had to be made. The Person Separation Index (PSI = 0.89) was excellent, indicating excellent discriminatory power between different levels of positive mental health. Overall, the targeting of the PMH-8 was good for the majority of the present sample. However, at both ends of the scale item thresholds are missing as indicated by a slight floor effect (1.4%) and a considerable ceiling effect (9.8%).

**Conclusions:**

Overall, the results of the analysis according to the Rasch model support the use of the revised PMH-scale in a psycho-oncological context.

**Supplementary Information:**

The online version contains supplementary material available at 10.1186/s12888-022-04162-0.

## Background

Mental health research has predominantly concentrated on psychopathology and symptoms [1]. In recent years, the focus from this deficit-centered approach started to change, taking into account the findings of positive psychology research and the recognition that mental health is not merely the absence of disease but rather a state of well-being that positively affects the whole range of life factors (e.g., coping with daily stressors and functioning in work and community) [2, 3]. Accordingly, facets of well-being, respectively positive mental health (PMH), and mental health problems, may be present simultaneously [4]. Attempts to conceptualize mental health assume that there are several PMH facets, which can be divided into eudaemonic well-being, i.e., positive psychological and social functioning in life, and hedonic well-being, i.e., positive emotions toward one’s life [5].

In psycho-oncology, the investigation of patient-reported outcomes and interventions likewise has mainly focused on psychological distress and quality of life [6]. Indeed, cancer regularly is associated with physical and mental distress. This distress depletes patients’ quality of life and negatively influences disease progression and survival rates [7–9]. However, research on well-being’s influence on mental health also shows effects and improves recovery and survival rates in physically ill patients [10]. Several psychological interventions like Acceptance and Commitment Therapy (ACT) [11] or well-being therapy [12] aim at enhancing well-being. Similarly, interventions for cancer patients like meaning-based interventions are rooted in positive psychology [13]. Importantly, positive mental health can help to protect cancer survivors against distress and demoralization [14].

This increased interest in positive mental health motivated the development of several assessment instruments [15]. Valid and reliable instruments are needed in order to be able to evaluate clinical interventions, to ensure sound clinical decision-making, and to select the most appropriate interventions for individual patients. To this end, a scale has been developed that combines the hedonic and the eudaemonic aspect of mental health [5] and aims to assess positive mental health with a brief, person-centered and unidimensional questionnaire [4]. Unidimensionality means that a scale primarily assesses one underlying construct. This is crucial because it ensures that the interpretation of the instruments’ scores is representative of the measured construct [16].

The PMH-scale is a self-rating questionnaire constructed to assess the positive dimension of the dual-factor model of mental health, i.e., integrating positive and negative mental health factors [17]. The scale is available in 12 languages and validated in a student sample, the general population, and a patient sample [4]. Usage is continuously increasing, for example, in research for predicting adaptive and maladaptive responses to the Coronavirus (COVID-19) [18], in studies looking at cross-cultural differences [19], and suicide ideation [20].

Several psychometric studies based on classical test theory (CTT) have been conducted using the PMH-scale. They generally demonstrated high internal consistency, good retest- reliability, good discriminant and convergent validity, and supported unidimensionality within samples of students, patients, and the general population (e.g., [4, 19, 21, 22]). However, in CCT based analyses, scores are calculated by summing up the responses on items and these test scores are assumed to be on interval scale level which is normally not the case [23]. An alternative to CCT is item response theory (IRT), which is a group of measurement models that explain the relationship between the responses to items and the person location of an underlying latent trait [24]. One of these modern approaches is the item analysis according to the Rasch model [25]. Since the measurement model is characterized by its simplicity, it occupies a special position among IRT models [26]. In case that person responses to scale items fit the Rasch model the ordinal score can be converted into an interval-level person parameter. There are numerous potential advantages of IRT models, including Rasch analysis, over CTT in assessing self-reported health outcomes. For example, it allows testing for unidimensionality, bias across different subgroups, and the systematic investigation of local dependency (LD) which might inflate the reliability of a scale. Additionally, it enables the examination of targeting and how the response options of items are used by the assessed persons. Focusing on individual items and how persons respond to those items allows for a more sophisticated analysis of the psychometric properties of the questionnaire under study [23, 24, 27, 28]. However, to the best of the authors’ knowledge, Rasch analysis has not yet been applied to the PMH-scale.

Since psycho-oncological interventions and cancer patients may benefit from positive effects of PMH improvement with respect to recovery and survival rates and as a protective factor, it is important to consider the PMH-scale application in the oncological context as well. However, research studying the psychometric properties of the PMH-scale in an oncological context does not yet exist. Against this background, we examined the psychometric properties of the PMH-scale in oncological context among various types of cancer patients using Rasch-analysis, especially to investigate the assumptions of unidimensionality, invariance across different exogenous variables, local independence of items. Additionally, a special focus was placed upon the investigation of targeting. A scale is well targeted to a sample if the majority of the sample is assessed with good measurement precision [29].

## Method

### Participants and procedure

Using SoSciSurvey [30], participants were invited to participate in the study as an online survey consisting of various questionnaires. Participants were asked about their cancer diagnosis and selected applicable types of cancer from a list. This question was designed as a multiple-choice task with several answer options as well as an open, descriptive category “other”, so that several cancer diagnoses could be named at the same time. Social media platforms, a forum for cancer patients, and mailing lists from self-help groups were used to advertise the study as part of another validation study [31]. All participants gave their informed consent online, after being informed about study content and aims, procedures, and planned publications. Inclusion criteria were: age ≥ 18 years and at least one current or past cancer diagnosis. No exclusion criteria were defined. In total, *N* = 357 cancer patients (*n* = 288 women (80.7%), *n* = 68 men (19.0%), *n* = 1 gender divers (0.3%)) completed the PMH-scale.

 The study was approved by the Ethics Commission of the University’s Faculty of Medicine (reference number 18–098). All procedures contributing to this work comply with the relevant national and institutional committees’ ethical standards on human experimentation and the Helsinki Declaration of 1975, as revised in 2008.

### Assessment instrument

PMH. The German version of the PMH-scale [4] was used, a self-report instrument consisting of nine items rated on a four-point rating scale ranging from 0 (“do not agree”) to 3 (“agree”). It assesses the emotional, psychological, and social indicators of positive mental health. Higher scores reflect greater positive mental health. In a series of six studies that included samples of students, patients, and the general population, the scale showed good psychometric properties e.g., high internal consistency (Cronbach’s alpha =. 93), satisfactory retest reliability (*r* = 0.74 − 0.81), and convergent validity was confirmed, e.g., with Satisfaction With Life Scale [32] (*r* = 0.75), Subjective Happiness Scale [33] (*r* = 0.81) [4], and demonstrated strong cross-cultural measurement invariance in student samples from Germany, Russia, and China [19].

### Statistical analyses

Data were analyzed using SPSS version 26.0 [34] and RUMM2030 software [35].

To assess the psychometric properties of the PMH-scale in an oncological context, item analysis according to the Rasch model was used. IRT models, including the Rasch model, can be used to analyze the psychometric properties of an instrument in detail because they focus on individual items and how people respond to those items. The probability of an item response is a function of the difference between person parameters and item difficulty parameters on the latent trait, which in this case is PMH [4]. ‘Easy’ PMH-items would be items that are already scored highly toward the positive health dimension by participants with only low PMH, whereas ‘difficult’ PMH-items would be items that are only scored highly by participants with many emotional, psychological, and social aspects of positive mental health.

Performing a Rasch analysis involves examining how well the data meet the expectations of the measurement model and whether certain requirements are met. This is a requirement of Rasch models, that the data must fit the model, not the other way around [36]. As with other IRT models, the requirements relate i.a. to unidimensionality, local independence, and the absence of differential item functioning (DIF). Specific to Rasch analyses is the requirement of homogeneity. The analysis of the Rasch model can be understood as an iterative process in which the model assumptions are checked and potential deviations found are resolved, if possible. Accordingly, whether the data fit the Rasch model or not depends on the evaluation of several different indicators as the consideration of the chi-squared item-trait interaction statistics, the item and person fit, the investigation of unidimensionality as well as of local independence and the absence of DIF. All these indicators will be described in more detail below. In case model fit is found, the transformation of ordinal scores into interval-level parameters is possible. The Rasch model uses a logistic transformation to convert ordinal scores into linear measures expressed in “logits” (i.e., log-odds units) [29].

Overall model fit, which evaluates the adequacy of the model for a data set as a whole, was evaluated using the chi-square item-trait interaction statistic. A good level of overall fit is characterized by a non-significant chi-square probability *p* > 0.01 [29, 37, 38]. To conclude a good fit, the mean values of the residuals should be around 0 and have a standard deviation of 1. Besides the overall fit, the fit of the individual items (item fit) and persons (person fit) can be evaluated and are expressed as residuals. The fit z-residuals are expected to be within a range of ± 2.5 [29, 39]. The second fit-statistic is a chi-square statistic and the chi-square probability should be non-significant.

One fundamental requirement of the Rasch model is unidimensionality, i.e., the items of a scale should capture only one underlying construct, which was tested with principal component analysis (PCA) of the residuals [29, 37]. The idea is to use the items with the highest negative/positive loadings on the first component to create two subsets of items. The separate person estimates of these two subsets are used to identify significant differences with independent *t*-tests. The proportion of significant *t*-tests should not exceed 5% to reject multidimensionality and infer unidimensionality [40].

Another assumption is that of local independence. This assumption implies that there should be no residual correlations between items when extracting the trait factor [41]. LD can occur when items are linked such that the response to one item determines the response to another item [37, 41]. Because LD can lead to overestimation of reliability, bias in parameter estimation, and corrupt construct validity [42] adequate handling of it is critical. Local independence was investigated using a residual correlation matrix of the items. Items with a residual correlation of 0.2 above the average were considered as locally dependent [42, 43]. One strategy to deal with LD if one does not want to delete scale items is to combine the locally dependent items into ‘superitems’. ‘Superitems’ are locally dependent items that are added to a larger and higher-ordered polytomous item that combines the scores of the locally dependent items. Using the ‘superitem’-strategy results in a bi-factor equivalent solution. The proportion of explained common variance (ECV) [44–46] of the general factor, should be > 0.9 to consider the scale as unidimensional [44].

A specific assumption of the Rasch model is that the items are assumed to be homogeneous in the sense that the ranking of the item parameters should be the same for all respondents, regardless of their expression of the latent trait. This requirement is reflected in tests of item-trait interaction based on group residuals, i.e., differences between observed and expected scores in groups matched by their total person-parameters scores [39, 41, 47].

Another assumption is the absence of DIF. If DIF is found, the difficulty of an item is different for different groups (e.g., men and women). In other words, the corresponding item indicates the latent trait in different ways in different groups [29, 41]. DIF analyses were examined using analysis of variance (ANOVA). Uniform DIF is shown by a significant main effect for person factor indicating that the different groups show a consistent difference in their responses to an item across the whole range of the assessed dimension. The presence of non-uniform DIF is shown by a significant interaction effect (person factor x class interval) indicating that the differences between groups vary across the levels of the assessed dimension. In this study, we tested the items for DIF in relation to gender (woman, man), age (median split of the sample: below and above 54), type of cancer (breast, other forms of cancer, multiple cancers), presence of metastases (yes, no, unknown), psycho-oncological support (yes, no) and duration of disease (median split of the sample: below and above 3.9 years). To avoid too small subgroups in the ANOVA, we had to exclude the one gender divers person, and the metastasis category ‘unknown’ from the DIF analysis and combine the remaining cancer diagnoses with lower frequencies into one category ‘other forms of cancer’ for the cancer type DIF analysis. In the case of DIF, several strategies to deal with can be used. One possibility is to remove or reformulate items with DIFs or to split the item with regard to the respective DIF-variable. We used the latter strategy and split the item in case DIF was found and subsequently evaluated the impact of DIF by computing equated scores [26]. Following this method, the item for which DIF was found, is split for the respective DIF-variable (e.g., for gender). For each DIF-subgroup (e.g., males vs. females) a score-to-measure transformation is performed and for each person parameter the equated scores of e.g., males and females can be compared and the size of score differences can be evaluated [48, 49].

Moreover, to assess the category functioning of each item, the threshold ordering was analyzed using the category probability curves. Item thresholds are the transition points between two adjacent response categories. Disordered thresholds can affect the interpretation and validity of scale scores [50]. There may be several causes of threshold disorder, such as respondents having difficulty to consistently differentiate among response options or LD causing the disorder. If the disorder is due to problems with category differentiation, one option is to collapse the disordered response categories together.

The reliability of the scale was estimated using the Person Separation Index (PSI). The PSI indicates the discriminatory power of how well a set of items can distinguish between the individuals being measured. PSI values of 0.7 are considered appropriate for group and 0.85 appropriate for individual applications [29, 37, 39, 41].

Targeting describes the extent to which a scale is appropriate for a given sample in terms of scale difficulty. Targeting was assessed graphically using the person-item threshold distribution graph. Person-item maps show how person parameters and item thresholds are distributed along the measured dimension [29]. They indicate whether the item thresholds are located in the same range as the person parameters. If a scale is poorly targeted for a sample, the measurement precision is low in those ranges of the assessed dimension in which the persons are located. In case of the PMH-scale the scale would be poorly targeted if respondents either report less well-being than the scale assesses or have a higher level of well-being. Additionally, the extent of floor and ceiling effects and a mean person parameter deviating substantially from zero (which usually is the mean value of the item difficulty) can be indicators of poor targeting [29, 41].

Due to the polytomous nature of the PMH items a derivation of the Rasch model for polytomous data had to be used. There are two different models which can be considered, in fact the Rating Scale Model (RSM) [51] and the Partial Credit Model (PCM) [52]. The difference between both models is that in the former the distances between adjacent thresholds are assumed to be equal across all items whereas in the latter the category breadth can vary across items. A likelihood ratio statistic can help to decide which model should be used.

## Results

### Sample

The mean age of the *N* = 357 participants was 52.40 years (*SD* = 14.01). All participants completed the PMH questionnaire. A selection of descriptive statistics and an overview of cancer diagnoses among the participants are presented in Table 1. The cancer diagnosis question was a multiple choice question, and some respondents (*n* = 46, 12.9%) reported more than one cancer diagnosis.

**Table 1:** Characteristics of cancer patients (*N* = 357)

Gender	
Male	68 (19.0)
Female	288 (80.7)
Divers	1 (0.3)
Age (years)	52.40 ± 14.01 (20–83)
Job situation	
Active	147 (41.2)
Certified sick	58 (16.2)
Different form	152 (42.6)
Types of cancer^a^	
Breast	162 (45.4)
Urological	31 (8.7)
Prostate, testicular	25 (7.0)
Gynecological	19 (5.3)
Hematological	22 (6.2)
Intestinal, rectal	12 (3.4)
Skin	3 (0.8)
Lungs, Bronchia	5 (1.4)
Ear, Nose, Throat	7 (2.0)
Gastric, esophageal, pancreatic	4 (1.1)
Parts of central nervous system	2 (0.6)
Soft tissue	1 (0.3)
Residual category (including different forms of cancer) ^b^	18 (5.0)
Multiple cancer forms	46 (12.9)
Metastases	
No	267 (74.8)
Yes	78 (21.8)
Unknown	12 (3.4)
Current psycho-oncological, psychological, psychotherapeutical support	
No	256 (71.7)
Yes	101 (28.3)
HADS-T Score - Distress (HADS T ≥ 15)	158 (44.3)

Values are presented in frequency (%) or mean±standard deviation (range). *HADS−T *Hospital Anxiety Depression Scale [53] (To identify patients with an increased need for psycho−oncological care and especially for depression symptoms in cancer patients, a sum score of HADS−T ≥ 15 can be used as the cut−off value) [54]; ^a^Patients reporting more than one type of cancer diagnosis are reported in the multiple cancer forms category; ^b^ Patients could also select the residual category “other tumor, and this is” and name the tumor in an open−ended question. Those tumors that did not fit into the above categories were included in the residual category, which included e.g., osteosarcomas, chondrosarcomas, and malignant neoplasms of the liver

### Analysis according to the Rasch model

In the initial analysis, we reviewed the model assumptions and determined how well the data met the expectations of the measurement model assessing several indicators, which are summarized below: The results of this initial analysis of all nine PMH items showed an unsatisfactory overall model fit (χ^2^ = 72.75, *p* = 0.005). The items statistics displayed misfit with a residual mean of -0.32 (*SD* = 2.57). For persons the residual mean was − 0.37 (*SD* = 1.23), indicating no serious misfit. Several pairs of items displayed LD, and DIF was found for item 5 (‘I manage well to fulfill my needs.’) in relation to age. Three items showed item-misfit when using the fit residual as criterion, but using the chi-square statistic and Bonferroni correction no significant item misfit was found. However, the p-value of item 9 (‘I am a calm, balanced human being.’; *p* = 0.007) was only slightly above the Bonferroni corrected significance level (*p* = 0.005) and had a very high fit residual (4.95), reflecting potential multidimensionality. We decided to exclude item 9 from further analyses. The other two misfitting items based on item fit residuals were items 3 (item residual = -2.79) and 8 (item residual = -2.54), which had too high negative item residuals indicating possible LD. All items showed ordered thresholds. Person misfit was negligible with only four patients (1.12%) showing fit residuals higher than 2.5. The test statistics of the nine PMH items of the first observation analysis are shown in Table 2, which shows the item location (difficulty), the corresponding standard errors (SE), the item residuals indicating the item fit, and chi-square statistics.

In the initial analysis it was found that the RSM should be favoured over the PCM as indicated by a non-significant likelihood ratio test. However, after modifications to the PHM-scale had been undertaken in subsequent analyses overall fit to the Rasch model was better when using the PCM.

**Table 2:** Initial analysis test statistic of the nine items of PMH-Scale (items ordered by location)

PHM-Scale items	Loc	SE	Res	χ2 (df)	*p*
**8) Much of what I do brings me joy.**	-0.96	0.10	-2.54	10.80 (5)	0.055
**2) I enjoy my life.**	-0.65	0.10	-1.31	2.65 (5)	0.754
**4) In general, I am confident.**	-0.57	0.10	-1.47	9.10 (5)	0.105
**3) All in all, I am satisfied with my life.**	-0.10	0.09	-2.79	7.88 (5)	0.163
**5) I manage well to fulfill my needs.**	0.06	0.09	-1.21	9.36 (5)	0.096
**7) I feel that I am actually well equipped to deal with life and its difficulties.**	0.12	0.09	-1.93	6.53 (5)	0.258
**1) I am often carefree and in good spirits.**	0.44	0.09	1.40	3.28 (5)	0.656
**9) I am a calm, balanced human being.**	0.67	0.09	4.95	15.81 (5)	0.007
**6) I am in good physical and emotional condition.**	1.02	0.09	1.99	7.36 (5)	0.195

*Loc (Location) *difficulty; *SE *Standard error, *Res (Residual) *item fit, *χ2 *Chi−square, *df *degrees of freedom, Bonferroni adjusted significance level: 0.005556

Based on this overall view, LD seemed to be the major problem, so we focused on accounting for it after excluding item 9 because of misfit. Starting with the highest residual correlation and adjusting successively for the following higher correlation and always checking model fit, item pairs 1&2, 3&4, and 6&7 were combined into ‘superitems’. After applying these strategies, there was no further evidence of LD nor of item or person misfit. The assumption of unidimensionality could be derived (significant *t*-tests: 4.10%). The ECV was 0.99, indicating a high explained common variance and also suggesting the scale’s unidimensionality as well. All items still showed ordered thresholds. The test statistics of the eight PMH items in the final analysis are shown in Table 3, which again shows the item location (difficulty), the corresponding standard errors (SE), the item residuals indicating the item fit, and chi-square statistics.

**Table 3:** Final analysis test statistic of the eight items of PMH-Scale (items/ ‘superitems’ ordered by location)

Items/ Super items	PHM-Scale items	Loc	SE	Res	χ2 (df)	*p*
**i5**	5) I manage well to fulfill my needs.	-0.79	0.10	-0.96	12.22 (5)	0.032
**s3_4**	3) All in all, I am satisfied with my life. 4) In general, I am confident.	-0.23	0.07	-0.69	2.23 (5)	0.817
**s1_2**	1) I am often carefree and in good spirits. 2) I enjoy my life.	0.07	0.07	1.10	1.41 (5)	0.922
**i8**	8) Much of what I do brings me joy.	0.23	0.10	0.16	11.07 (5)	0.050
**s6_7**	6) I am in good physical and emotional condition. 7) I feel that I am actually well equipped to deal with life and its difficulties.	0.73	0.06	1.53	3.41 (5)	0.637

*Loc (Location) *difficulty, *SE *Standard error, *Res (Residual) *item fit, *χ2 *Chi−square, *df *degrees of freedom, Bonferroni adjusted significance level: 0.01000

In the final analysis, there was no DIF in relation to gender, type of cancer, presence of metastases, psycho-oncological support and duration of disease. However, uniform DIF related to age was found for ‘superitem’ 1&2 (*p* = 0.001) and item 5 (*p* < 0.001) (see Table 4). The DIF found initially suggests that elderly individuals seem to find it easier to meet their needs than younger individuals with the same level of well-being (item 5), and younger individuals seem to find it easier to enjoy life than older individuals with the same level of well-being (‘superitem’ 1&2).

**Table 4:** DIF summary (Age) of the eight items of PMH-Scale

Items/ super items	Uniform DIF for Age				Non-Uniform DIF for Age			
	M	F	DF	Prob	M	F	DF	Prob
s1_2	9.46	10.74	1	*0.001*	0.14	0.16	5	0.978
s3_4	0.09	0,12	1	0.730	2.19	3.06	5	0.010
s6_7	1.44	1.58	1	0.210	1.66	1.82	5	0.108
i5	9.80	12.87	1	*0.000*	0.18	0.23	5	0.948
i8	0.30	0.43	1	0.512	0.56	0.80	5	0.547

Bonferroni adjusted significance level: 0.003333. Items showing significant p−values marked in italics

We investigated the impact of the found DIF with the before mentioned methods. After splitting item 5 for age-DIF, there was no more evidence of age-related DIF for ‘superitem’ 1&2 (*p* = 0.840) indicating that the latter was probably artificial DIF [55]. To evaluate the magnitude of the found age-related DIF in item 5, equated scores were computed. The difference in the equated scores between the younger and older patients was only minor, with a maximum score difference of about 0.5 points in the lower range of the PMH dimension (between − 4 and − 3). However, in the other parts of the dimension, the difference was even more negligible. The equated scores are presented in Table 5. Thus, as the age-DIF was considered as being not substantial, we decided not to split this item for age in the final solution.

**Table 5:** Equated scores showing the minor impact of age-DIF

Age < 54	Age ≥ 54
**1**	1.50
**2**	2.47
**3**	3.43
**4**	4.38
**5**	5.33
**6**	6.29
**7**	7.26
**8**	8.25
**9**	9.26
**10**	10.29
**11**	11.32
**12**	12.35
**13**	13.37
**14**	14.36
**15**	15.33
**16**	16.29
**17**	17.22
**18**	18.14
**19**	19.06
**20**	19.97
**21**	20.88
**22**	21.79
**23**	22.69
**24**	23.41

The final solution’s overall model fit with eight items was satisfactory (χ^2^ = 30.34, *p* = 0.21) with excellent reliability PSI = 0.89. After these adjustments, no patient showed fit residual scores higher than 2.5. The summary test statistics of the initial and final analyses are presented in Table 6 with the number of items, overall model fit, unidimensionality test, reliability, item and person fit (residuals), and item misfit.

**Table 6:** Overall summary of test statistic

	# items	Item-Trait interaction (Overall fit)			Uni-dimensionality t-test	Reliability	Item-Residual		Person-Residual		Item misfit
Analysis		**χ**^**2**^	**df**	***p*** **-value**	**test (%)**	**PSI**	**M**	**SD**	**M**	**SD**	**Item number**
Initial	9	72.75	45	0.01	3.31	0.90	-0.32	2.57	-0.37	1.23	3, 8, 9
Final	8	30.34	25	0.21	4.10	0.89	0.23	1.09	-0.32	1.01	None

* df* degrees of freedom, *# items *number of items, *Item−/ Person Residual *differences between observed and expected responses, *M *Mean, *PSI *person separation index, *SD *standard deviation, *χ2 *Chi−square

**Fig. 1:**
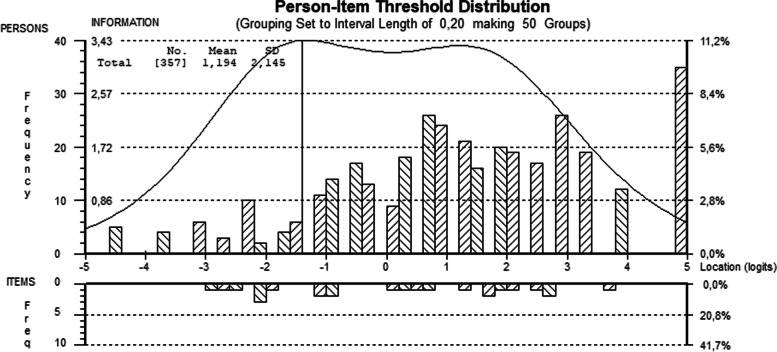
Person-Item threshold distribution (final analysis). Note. Person-item threshold distribution of the PMH responses. Higher values indicate a higher level of well-being (top of the half) and higher item difficulty (bottom half). At the left side the frequency and at the right side the percentage of persons respectively items are displayed

Figure 1 shows the targeting of the scale. Overall, the item threshold distribution shows that the scale measures a wide range of positive mental health, except for very low levels and very high well-being levels. The majority of the patients of the present sample were located within the same range as the item threshold parameters. The mean person location value was *M =* 1.19 (*SD* = 2.15). This value means that the patients had a slightly higher level of well-being than the scale’s center (which is 0). Thus, the person distribution demonstrates slight mistargeting, with more people showing higher levels of well-being and 9.8% of people having the highest possible score (ceiling effect). There were also a few persons with the lowest possible score (1.4%) (floor effect).

## Discussion

This study is the first to provide information on the psychometric properties of the PMH scale within a sample of cancer patients and the first to use a modern psychometric analysis, i.e., Rasch analysis with its many potential advantages over CTT in assessing self-reported health outcomes. The use of relevant and cancer-specific DIF variables in this study should be highlighted. Adequate interval level measurement is of great importance when evaluating clinical interventions, ensuring sound clinical decision-making, and monitoring changes across the course of treatment.

Especially interventions for improving PMH for cancer patients like ACT or meaning-based interventions in psycho-oncology can reduce mental health problems and have positive effects on recovery and survival rates [10, 14]. Assessing the current status of the PMH of patients can be a starting point for selecting appropriate interventions for patients.

Overall, the PMH-scale showed a good model fit and excellent reliability after making some modifications due to LD and excluding one item. The excluded item was item 9, which displayed an item residual of 4.95. In contrast to our study, this item showed adequate factor loading in CTT studies [4, 19], even though it had by far the smallest loadings. Compared in context to the other items of the scale, it appears that item 9 (‘I am a calm, balanced human being’) assesses two different aspects. One can be hectic but still be balanced (i.e., exhibit positive mental health). Moreover, it seems to reflect trait character to a higher degree than the other items. According to the Rasch model, this trait character could be the reason why item 9 was misfitting in our analysis. Further research in other samples is needed to further investigate the fit of this item.

Furthermore, the scale contained several pairs of locally dependent items. After combining the locally dependent item pairs successively into ‘superitems’, no more LD was observed. In terms of content, the observed LD within the scale makes sense since items 1&2 are facets of enjoying life, items 3&4 assess satisfaction in the present and future, and items 6&7 are concerned with mastering daily life. In a study with a cross-cultural sample, a similar dependence was found between Item 1 and Item 2, and the same conclusion was drawn that these items relate to facets of enjoyment of life [19]. Since there are no other studies using Rasch analysis, future studies should also focus on investigating LD in the PMH-scale, given the influence of LD on parameter estimation and reliability.

DIF was tested in relation to gender, age, type of cancer, the presence of metastases, psycho-oncological support, and duration of disease. For most of these external variables, no DIF was found. However, uniform age-DIF was found for ‘superitem’ 1&2 and item 5. As the DIF for ‘superitem’ 1&2 was no longer present after splitting item 5 for DIF related to age, this might indicate that this DIF was artificial [55]. To evaluate the impact of the age-related DIF found for item 5, equated scores were computed. We only found a relatively small inconsiderably difference in the equated scores between the younger and older patients, with a maximum score difference of about 0.5 points in the lower range of the person location. This result shows an indication that patients with the same level of well-being responded differently to the managing to fulfill their needs item depending on their age. Specifically, elderly individuals seemed to have more ease in this field than younger persons with the same well-being level. However, this difference becomes visible only in the lower range. In contrast, patients with either a high or middle level of well-being responded comparable in the areas of high or middle level of well-being, irrespective of their age. Given the minor impact of DIF and given that it was only found in a tiny part of the assessed dimension, we decided not to adjust for DIF. Note that our sample is relatively young, with a mean age of 52.40 years. In a sample with more elderly patients, a more relevant age-DIF might be found.

The conclusion on unidimensionality is consistent with other CTT analyses of the PMH-scale [4, 19, 21]. Overall, the targeting of the PMH-8 scale was good for the present sample of cancer patients. The PMH showed a widespread distribution of item thresholds that ensured good measurement accuracy across a large portion of the PMH dimension. However, for low and high PMH levels, the targeting was not as good as item thresholds were missing in these areas of the dimension. The PMH-scale was initially developed to provide a unidimensional assessment of PMH in the general population. Our results indicate that the differentiation in the higher segment of well-being is not equally good – an area where probably most of the people of a healthy population would be located. However, the differentiation within a healthy population or persons with a high, respectively a very high level of PMH may not be so relevant for the assessment of oncology patients with regard to clinical decision-making in psycho-oncology. Easier items are also missing, making it also hard to precisely assess PMH at a low level of well-being. It might be attractive in future research to either include some more items or to develop a better targeted scale for patients with low levels of well-being (e.g., with items related to other facets of mental health like life affirmation or meaning of life). This potential revision could be used, for example, to have a first starting point for resource-activation work with patients in psycho-oncological interventions. However, given the heterogeneity of individuals and their variability in perceiving the benefits of an intervention and their response to it, it is critical to identify individual variation in clinical significance of change in health care. Therefore, concepts of clinical significance of change are increasingly being used to improve change measurement and clinical decision making. Future studies should consider the clinical significance of the scale by also examining its use in the clinical setting based on individual significance.

Besides some strengths, the present study also has some limitations. The sample consisted of a relatively high percentage of breast cancer patients. The residual cancer types had to be combined into one category, ‘other forms of cancer’ for the DIF analysis due to small subgroup sizes. Accordingly, the results may only be generalized to other cancer patients with caution. Future studies with larger samples and higher proportions of different cancer types should be investigated, especially with regard to gender-specific cancer diagnoses and thus a possible gender DIF. However, in our analysis, we found no evidence of a gender-DIF. Future research is also needed regarding the influence of different cancer types, especially those with a more severe disease progress. Nevertheless, the presence of metastases or the disease duration could also be used as an indication of severity. We examined both in our study, and both of them showed no DIF. Furthermore, the DIF analysis with cancer types was included because, in addition to breast cancer, reporting multiple cancer types could be an indicator of more severe disease. Additionally, the sample’s psychological distress (HADS-T) is roughly equally distributed across the cancer forms. Therefore, one can assume that the type of cancer does not unduly influence the response behavior. Furthermore, the recruited sample is relatively young, with a mean age of 52.4 years. This may be the result of the recruitment procedure. The sample was recruited from social media platforms and from online cancer support groups. The scale assesses a wide range of well-being, but for the present sample it shows a slight mistargeting and an off-center distribution of persons with a relatively high frequency of persons with a high PMH level, which may indicate a bias in this sample. Also, a high percentage (41.2%) of the cancer patients had an active job situation, indicating a relative fit sample. Concerning this and the small age-DIF we found in our study, future research should examine a sample with a lower level of mental health and perhaps include some additional items suited for assessing lower and higher levels of PMH.

## Conclusions

The present study provides basic information about the psychometric properties of the PMH-scale in the oncological context. The Rasch analysis showed that this scale can be used well in this context; in particular, it adequately captures individuals with intermediate PMH scores. However, the scale should be further investigated for its targeting, and better targeted items may need to be added to capture the full range of the PMH dimension. Given that PMH can predict mental health problems and positively impact recovery and survival rates, these findings are useful, especially for selecting appropriate interventions for patients. The instrument is non-biased with respect to gender, type of cancer, the presence of metastases, psycho-oncological support, and duration of disease. However, with regard to age, especially in elderly people, a critical consideration might be necessary.

## Supplementary Information

**Additional file 1.**

## Data Availability

Data not published within the article can be shared upon reasonable request from alexander.gerlach@uni-koeln.de.

## Abbreviations

ACT: Acceptance and Commitment Therapy
ANOVA: Analysis of Variance
CTT: Classical Test Theory
DIF: Differential Item Functioning
GSE: General Self-Efficacy
HADS: Hospital Anxiety and Depression Scale
HADS-T: HADS sum score
IRT: Item Response Theory
LD: Local Dependence
PCA: Principal Component Analysis
PCM: Partial Credit Model
PMH: Positive Mental Health
PSI: Person Separation Index
RSM: Rating Scale Model
