# Supplementary material for: Psychometric evaluation of the Positive Mental Health (PMH) scale using item response theory
Source: BMC Psychiatry. 2022 Jul 28;22:512. doi: 10.1186/s12888-022-04162-0 (PMC9334023; doi:10.1186/s12888-022-04162-0)
Supplement: Supplementary file 1 — Additional file 1. [file 12888_2022_4162_MOESM1_ESM.docx]

Supplementary Table 1 Conversion table of the PHM-8 scale

| PMH ordinal scale score | Interval-scaled person estimate | Transformed interval  scale 0–100 |
| --- | --- | --- |
| 0 | -4.54 | 0 |
| 1 | -3.70 | 9 |
| 2 | -3.10 | 15 |
| 3 | -2.67 | 20 |
| 4 | -2.32 | 24 |
| 5 | -2.02 | 27 |
| 6 | -1.73 | 30 |
| 7 | -1.45 | 33 |
| 8 | -1.17 | 36 |
| 9 | -0.88 | 39 |
| 10 | -0.58 | 42 |
| 11 | -0.27 | 46 |
| 12 | 0.04 | 49 |
| 13 | 0.36 | 52 |
| 14 | 0.67 | 56 |
| 15 | 0.97 | 59 |
| 16 | 1.26 | 62 |
| 17 | 1.55 | 65 |
| 18 | 1.84 | 68 |
| 19 | 2.15 | 71 |
| 20 | 2.48 | 75 |
| 21 | 2.85 | 79 |
| 22 | 3.31 | 84 |
| 23 | 3.95 | 91 |
| 24 | 4.83 | 100 |
